# Undiagnosed HIV Presenting with Lymphoid Interstitial Pneumonitis

**DOI:** 10.1155/2011/246706

**Published:** 2011-10-12

**Authors:** Jason J. Rizqallah, Christopher T. Shah, Oladoyin Oluwole, John N. Sheagren

**Affiliations:** College of Human Medicine, Michigan State University, 15 Michigan Street NE, Grand Rapids, MI 49503, USA

## Abstract

Undiagnosed or untreated human immunodeficiency virus infection can lead to devastating complications. We present a case of a 41-year-old woman who was found to have HIV-related lymphoid interstitial pneumonitis. LIP is uncommon, and its presentation can be quite similar to that of other chronic lung conditions. This case illustrates one of the possible protean manifestations of untreated HIV and is a sobering reminder of the need to screen all adults for HIV infection. Additionally, further invasive diagnostic testing may be required to guide therapy in patients with advanced acquired immune deficiency syndrome. This patient's LIP was likely related to long-standing unrecognized HIV disease.

## 1. Background

A 41-year-old woman with self-diagnosed asthma presented to the emergency department with progressive shortness of breath and productive cough of a one-year duration. She was of African American descent. Her shortness of breath was initially present only during periods of exertion. However, at the time of presentation, she reported that she was out of breath even at rest. She had paroxysmal coughing spells which occurred about four times per day for several minutes. During these spells, she was producing a moderate amount of yellow sputum but denied hemoptysis. She also admitted to orthopnea, a 15-pound unintentional weight loss over one year, and posttussive emesis. She had worsening pleuritic chest pain on the day of admission which prompted her to go to the hospital. 

She had a history of approximately 2-3 cigarettes per day for the last 20 years, although her last cigarette was five and a half months ago. She denied any illicit drug or alcohol use. She reported having two male sexual partners in her lifetime, the last of which was 4-5 years ago. She cannot recall whether or not she used protection at that time. She stated she had been working in a warehouse that deals with newspapers for about nine years and that she worked with paper dust as well as fumes. She denied the use of appropriate protective equipment at work and did state that her symptoms did not improve when she left the work environment.

The patient had not been seen by a physician in approximately 20 years until she presented to a different hospital two years ago for symptoms of bilateral chest pain. At that time she stated the duration as one year with associated tachypnea and tachycardia. She also stated that she had been living in a home with “mold and mushrooms.” Physical exam at that hospital revealed bilateral inspiratory and expiratory wheezes and rales. She had a normal chest X-ray, received albuterol and ipratropium nebulizers, IV ceftriaxone and methylprednisolone, and was discharged. She was eventually lost to followup due to lack of insurance.

## 2. Objective

Pertinent physical findings during her most recent admission included oxygen saturation of 97% on 3 L of O_2_ and 81% on room air. The patient was a cachectic-appearing woman who was noted to have temporal muscle wasting upon examination. A prominent submental node was palpable. However, the patient insisted this had been present for her entire life. Cervical/axillary lymphadenopathy was not noted on her physical exam. She had bilateral crackles as well as inspiratory wheezes and squeaks, more prominent in the upper lobes of the lungs. Finally, she had digital clubbing present in her upper extremities. Initial laboratory findings were white-cell count, 3.9 × 10^3^/*μ*L; hemoglobin, 10.1 g/dL; hematocrit, 30.7%; mean corpuscular volume, 80.1 *μ*m^3^; sodium, 136 mEq/L; potassium, 4.4 mEq/L; chloride, 105 mEq/L; bicarbonate, 28 mEq/L; brain natriuretic peptide, 231 pg/mL. Liver function tests were normal, as were her urine analysis and fecal occult blood test. An echocardiogram revealed right ventricular hypertrophy.

## 3. Methods and Findings

**Figure 1:**
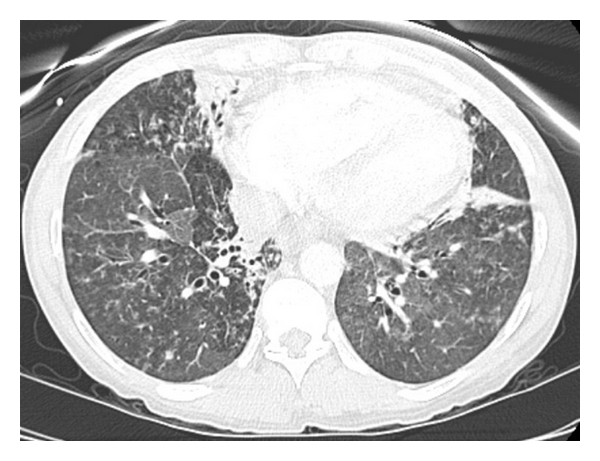
Computed tomography of the thorax showing extensive nodular, reticular, and groundglass opacities in both lungs along with mediastinal enlargement.

A radiograph of the chest revealed a new, diffuse, bilateral parenchymal opacity which appeared to have a mixed interstitial and airspace appearance. Computed tomography (CT) of the thorax revealed extensive irregular nodular opacities intermixed with areas of reticular opacity and groundglass opacities (Figure 1). These findings were widespread throughout both lungs, but more concentrated in the upper lobes. There was mediastinal and bilateral hilar lymph node enlargement, which was best appreciated on intravenous contrast images. Finally, there were small, mildly enlarged bilateral axillary lymph nodes. The findings appeared to be most consistent with sarcoidosis.

To refine our differential at this point we ordered the following studies: serum ACE levels to evaluate for sarcoidosis, ANA and rheumatoid factor, sputum cultures, urine histoplasma antigen, serum histoplasma, blastomyces, and toxoplasmosis antibodies, and serum human immunodeficiency virus (HIV) 1 and 2 antibodies. In addition, the patient was scheduled for a bronchoscopy with bronchoalveolar lavage (BAL) as well as transbronchial biopsy with needle aspirate. BAL showed no evidence of malignancy. The *Pneumocystis jiroveci* (formerly *Pneumocystis carinii* pneumonia or PCP) direct fluorescent antibody (DFA) assay was negative. Pathological specimens obtained from the right middle lobe of the lung showed a bronchial wall with chronic interstitial inflammation but no evidence of granulomatous disease. Fine-needle aspiration revealed minimal cellularity composed of rare degenerating epithelioid cells and scattered lymphoid cells. Sputum cultures grew *Pseudomonas aeruginosa* for which the patient was placed on levofloxacin. All other laboratory tests were negative except for serum HIV 1 and 2 antibodies which came back positive on both ELISA and western blot. Despite a negative DFA for PCP from bronchoscopy washings, there remained concern for PCP pneumonia. Therefore, the patient was started on trimethoprim-sulfamethoxazole and the dose of prednisone was slowly increased. Antiretroviral therapy was delayed to avoid immune reconstitution syndrome.

Owing to its high sensitivity and specificity, bronchoscopy with BAL is regarded as the gold standard for PCP diagnosis in HIV-1-infected patients [1]. However, based on the patient's clinical findings and CT images the possibility of a false negative remained a concern. The possibility of lymphocytic interstitial pneumonitis (LIP) was raised at this time—in which case it may have been more appropriate to have started the patient on highly active antiretroviral therapy (HAART) therapy which has been shown to be beneficial in selected cases [2–4]. The response, however, of HIV-associated LIP to intervention with antiretroviral therapy is variable. The risk of immune reconstitution inflammatory syndrome by treating with antiretrovirals before treating a previously acquired opportunistic infection such as PCP outweighed the need to start antiretroviral therapy [5].

To obtain a more definitive diagnosis, cardiothoracic surgery was consulted at that time and the patient was scheduled for video-assisted thorascopic surgery. Video thorascopy revealed a grossly abnormal-appearing left lung, with multiple nodules prevalent throughout the entire lung. Biopsies of the left lower lobe superior segment and left upper lobe lingual segment were obtained and sent for pathologic analysis.

**Figure 2:**
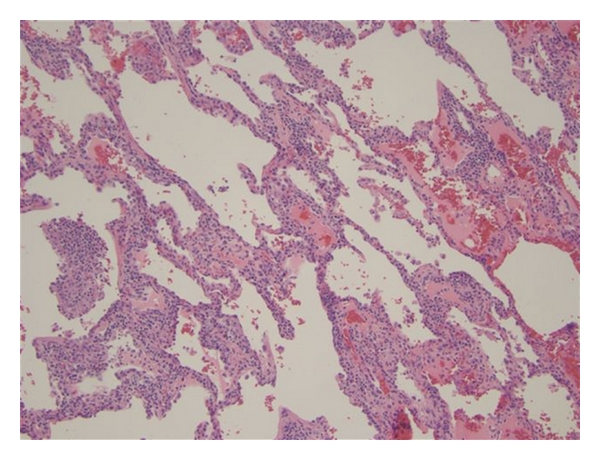
Haematoxylin and eosin stain of the left lobe biopsy showing extensive interstitial infiltrates and germinal centers.

**Figure 3:**
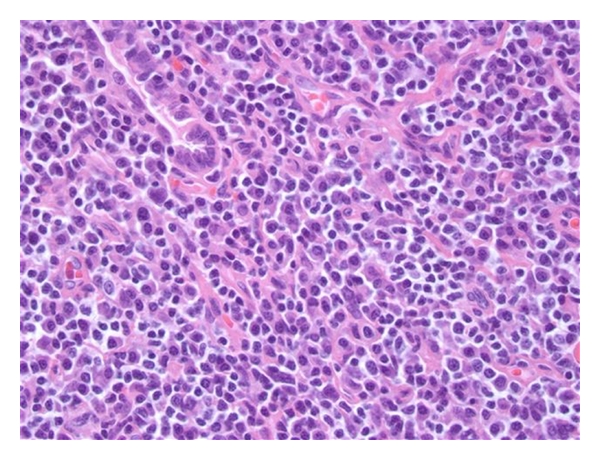
×40 magnification of left lung nodularity at a germinal center. Note the appearance of lymphoplasmacytic infiltrates with reactive appearing plasma cells.

Left lower and upper lobe biopsies showed extensive interstitial, nodular, and peribronchial lymphoplasmacytic infiltrates with reactive appearing plasma cells, including atypical plasma cells. In the nodular areas, the infiltrate contained germinal centers (Figures 2 and 3). Well-formed granulomas were not identified and there was no evidence of necrosis or angiocentricity. Given the polymorphous nature of the lymphoplasmacytic infiltrate, this was thought to represent a reactive/inflammatory process, specifically lymphoid interstitial pneumonia, which is seen in HIV infection, rather than a neoplastic process. Properly controlled immunostains for CD20 and CD3 demonstrated a predominance of CD3 positive T cells, also favoring a reactive lymphoid process. In addition, in situ hybridization for kappa and lambda demonstrated a polyclonal plasma cell population, and Ebstein Barr virus-encoded RNA in situ hybridization was negative.

## 4. Discussion

Recognizing potential presentations of patients with HIV, as evidenced by this case, can prove to be quite challenging especially early on in the course. HIV-associated LIP is relatively uncommon, occurring in less than 5% of adult necropsy case series [6, 7]. There appears to be a predilection in the adult population of HIV-associated LIP for those of African or Afro-Caribbean decent [6, 8]. The most common symptoms of LIP include progressive exertional dyspnea of several weeks' duration and nonproductive cough. Patients may also present commonly with fever, weight loss, and fatigue [6]. Clubbing is commonly seen in the pediatric population [9] and has been described sporadically in the adult population, even in the absence of bronchiectasis [10].

It is important to note that the diagnosis was overlooked upon the patient's initial presentation at another hospital. A mean time from initial presentation to diagnosis can exceed 15 months [11]. Based on the patient's living conditions reported at that time, her occupational exposure, her history of smoking, and the clinical similarities between her presentation and other common causes of dyspnea (especially those linked with her occupational and social risk factors), it was most likely considered that the patient had a lower respiratory infection overlapping an already diseased lung. With hindsight, it would have been recommended at the time of initial presentation to obtain a more detailed history, including drug and sexual history, and a complete workup including CBC, CMP, urine analysis, ECG, and testing for sexually transmitted infections considering the patient had not seen a physician in over 20 years.

After a nine-day course in the hospital, the patient was discharged on home O_2_, prednisone, trimethoprim sulfamethoxazole (after it was determined the patient had a CD4 count of 151 cells per uL), and ATRIPLA (600 mg efavirenz, 300 mg tenofovir, and 200 mg emtricitabine). The patient presented approximately five weeks later with shortness of breath, wheezing, productive cough, and fever of a one-week duration. She had also experienced a presyncopal episode the day before admission and had a fall at her home before arriving to the hospital. The patient was found to have *Streptococcus pneumoniae* bacteremia which was believed to be secondary to community-acquired pneumonia, was placed on piperacillin-tazobactam and vancomycin, and eventually discharged after her symptoms improved. She is currently following up with the local HIV clinic.

In summary, our case highlights the need for increased screening of HIV in the adult population, and especially in those who are not regularly following up with a primary care provider. Additionally, HIV-associated LIP should be included in the differential diagnosis of patients presenting with chronic cough and dyspnea who have risk factors for HIV. Implementation of these measures in the future may help physicians diagnosis HIV earlier in its course and lead to decreased patient morbidity through faster recognition and treatment of LIP.

## Abbreviations

CT: Computed tomography
HIV: Human immunodeficiency virus
BAL: Bronchoalveolar lavage
PCP: Pneumocystis carinii pneumonia
DFA: Direct fluorescent antibody
LIP: Lymphocytic interstitial pneumonia
HAART: Highly active antiretroviral therapy.
